# Study on the sleep quality of women pregnant with a second child and the influencing factors

**DOI:** 10.1186/s40001-022-00848-z

**Published:** 2022-10-17

**Authors:** Yi-Min Cai, Xia-Li Zheng, Zhou-Min Shen, Bi-Fang Zhou, Yu-Ming Liu, Jia-Yu Yang, Nian Xie

**Affiliations:** 1 Department of Nursing, Hunan Provincial People’s Hospital (The First Affiliated Hospital of Hunan Normal University), Changsha, 410005 China; 2 Department of Pediatrics, Hunan Provincial People’s Hospital (The First Affiliated Hospital of Hunan Normal University), Changsha, 410005 China; 3 Department of Interventional Vascular Surgery, Hunan Provincial People’s Hospital (The First Affiliated Hospital of Hunan Normal University), Changsha, 410005 China; 4 Department of Critical Care Medicine, The Second Xiangya Hospital, Central South University, Changsha, 410011 China

**Keywords:** Second child, Sleep quality, Pittsburgh Sleep Quality Index, Influencing factors

## Abstract

**Objectives:**

To investigate the sleep quality of women pregnant with a second child and the influencing factors and provide a scientific basis for health care guidance to clinically improve the sleep quality of pregnant women.

**Methods:**

A total of 162 women pregnant with a second child at a first-class tertiary hospital in Changsha from January to August 2018 were selected as the research subjects. General demographic characteristics were collected, and the Pittsburgh Sleep Quality Index (PSQI) scale was used to assess their sleep quality. Multivariate logistic regression analysis was used to explore the influencing factors of the sleep quality of women pregnant with a second child.

**Results:**

The PSQI score (except hypnotic drugs) and total score of pregnant women with second birth were higher than those of normal female population, and the difference was statistically significant (*P* < 0.05). Univariate analysis showed statistically significant differences in sleep quality among women pregnant with a second child of different ages, marital relationships, gender expectations, education levels, monthly family incomes, planned or unplanned pregnancy, and gestational weeks (*P* < 0.05). The results of the multi-factor analysis showed that the low education level (OR = 0.224, *P* = 0.001), low family monthly income (OR = 3.035, *P* = 0.014), expectation of gender (OR = 0.065, *P* = 0.038), and dissatisfaction with marital relationship (OR = 0.275, *P* = 0.001) were the primary risk factors of poor sleep quality of in women with second pregnant.

**Conclusions:**

The overall sleep quality of women pregnant with a second child is poor, and 37.65% have sleep quality problems. Low education levels, low family monthly incomes, fetal gender expectations, and poor relationship between husband and wife are the main factors affecting the sleep quality of women pregnant with a second child. Pregnant women with the above factors should pay attention to their sleep quality and take necessary measures for intervention and guidance to improve the level of health care during pregnancy.

## Introduction

Sleep is an essential life activity for human health [1]. Due to hormones, pregnant women have great changes in all systems of the body, which is easy to produce symptoms, such as fatigue and sleepiness. Studies have shown that the incidence of sleep difficulties in pregnant women is generally high, and there are significant problems in the sleep quality of pregnant women from different countries and regions [2]. The sleep status of pregnant women is not only related to their own health, but also affects the growth and development of the fetus. Relevant research reported that sleep disorders in pregnant women are closely related to pregnancy complications, such as preterm delivery [3], pregnancy induced hypertension [4], gestational diabetes mellitus [5] and postpartum depression [6]. The poor sleep quality in late pregnancy may lead to prolonged labor and increased cesarean section rate [7]. Therefore, it is particularly important to pay attention to the sleep quality of pregnant women.

Since the full opening of China's second child policy in 2016, women of childbearing age have increased their willingness to have a second pregnancy [8, 9]. Women pregnant with a second child are generally older and face pressure from family life, career, and even social life. They may also be more likely to have undesirable emotions, such as negativity, anxiety, and depression, affecting their sleep quality [6, 10]. The purpose of this study is to understand the current status of the sleep quality of women pregnant with a second child, explore the factors affecting their sleep quality, and provide theoretical support and guidance for clinicians to help them improve their sleep quality.

## Materials and methods

### Clinical data

A total of 162 women pregnant with a second child who attended the obstetric outpatient clinic or were hospitalized at the ward of a first-class tertiary hospital in Changsha from January to August 2018 were selected as the research subjects. The inclusion criteria: ① women diagnosed as pregnant with a second child. ② Informed consent and voluntary participation in this research. ③ No abnormal mental state, with normal expression and understanding abilities, barrier-free communication, and ability to fill out simple questionnaires independently. Exclusion criteria: ① severe infections during pregnancy, fetal malformations, twins, or multiple births. ② Women with acute, critical illness and severe pregnancy complications. ③ Patients with a history of mental illness and cognitive impairment. ④ Those who have language communication barriers and cannot communicate normally. This study was conducted in accordance with the Declaration of Helsinki and approved by the Ethics Committee of the Hunan Provincial People’s Hospital. All participants signed informed consent.

### Methods

The general information survey questionnaire and the Pittsburgh Sleep Quality Index (PSQI) questionnaire were used for this research. The questionnaires were sent out without delay and returned promptly, with a response rate of 100.00%. ① The general information questionnaire included age, occupation, marital status, education level, place of residence, economic income, marital relationship, family support (parents), gender expectation, planned or unplanned pregnancy, number of pregnancies, pregnancy complications, gestational week, history of miscarriage, couple relationship. ② The PSQI scale was compiled by Buysee et al. [11] in 1989. It contained 18 self-evaluation items, which were divided into 7 factors: subjective sleep quality, sleep latency, sleep time, sleep efficiency, sleep disorders, hypnotic drugs application and daytime dysfunction. The total score was 0 ~ 21 and PSQI < 7, the sleep quality is good; PSQI ≥ 7, poor sleep quality. The higher the total score, the worse the sleep quality. The sensitivity and specificity of the scale were 98.3% and 90.2%, respectively. It had good internal consistency, test–retest reliability, conceptual validity and empirical validity. The Pittsburgh Sleep Index abroad is widely used in the epidemiology of sleep disorders and the mechanism of sleep disorders, and has been shown to have good reliability and validity in multiple categories of populations [12, 13]; the Chinese version of the scale was revised by Liu Xianchen [14] and has been shown to have good reliability and validity [15].

### Statistical methods

Data from this study were analyzed using SPSS 19.0 statistical software. Shapiro–Wilk method was used to test the normality of measurement data, mean ± standard deviation (x ± s) was used for statistical description of measurement data, and *t* test was used for comparison between groups. Enumeration data were described using percentages, and comparisons between groups were performed using the *X*^2^ test. Logistic regression analysis was used for multivariate analysis. *P* < 0.05 was considered statistically significant.

## Results

### Sleep quality of women pregnant with a second child

The 162 women with second pregnancies in this survey were 23–42 years, with an average age of 31.92 ± 2.97 years. Among the 162 women pregnant with a second child, 61 had sleep quality problems (PSQI total score > 7 points), accounting for 37.65%. Of the seven areas of sleep quality, subjective sleep quality, daytime dysfunction, and time to fall asleep had the highest detection rate. Subjective sleep quality problems (> 1 point) accounted for 82.72%, daytime dysfunction (> 1 point) accounted for 79.01%, time to fall asleep (> 1 point) accounted for 69.14%, and the detection rate of hypnotic drugs was 0, see Table 1 for details.

**Table 1:** PSQI scores of second-born pregnant women

Item	Scope	Score	Number of cases (*n*)	Percentage (%)
Time to fall asleep	0–3	> 1	112	69.14
Sleep efficiency	0–3	> 1	18	11.11
Sleeping time	0–3	> 1	25	15.43
Subjective sleep quality	0–3	> 1	134	82.72
Sleep disorder	0–3	> 1	68	41.98
Daytime dysfunction	0–3	> 1	128	79.01
Hypnotic drugs	0–3	> 1	0	0
PSQI total score	0–21	> 7	61	37.65

The scores of all domains (except hypnotic drugs) and the total score of PSQI in pregnant women with second birth were higher than the normal female population norm, and the difference was statistically significant (*P* < 0.05), see Table 2 for details.

**Table 2:** PSIQ test results of pregnant women with second birth compared with normal female population (x ± s)

Item	Second-born pregnant women (*n* = 162)	Normal female [16] (*n* = 10,373)	*t* value	*P* value
Time to fall asleep	1.19 ± 0.63	0.80 ± 0.98	5.049	< 0.001
Sleep efficiency	0.65 ± 0.84	0.50 ± 0.95	1.997	0.023
Sleeping time	0.74 ± 0.68	0.37 ± 0.70	6.679	< 0.001
Subjective sleep quality	1.38 ± 0.96	0.83 ± 0.70	9.857	< 0.001
Sleep disorder	0.94 ± 0.57	0.36 ± 0.52	14.065	< 0.001
Daytime dysfunction	1.31 ± 0.82	0.48 ± 0.82	12.784	< 0.001
Hypnotic drugs	0	0.11 ± 0.51		
PSQI total score	6.21 ± 2.46	3.45 ± 3.70	9.461	< 0.001

### Univariate analysis of factors affecting the sleep quality of women pregnant with a second child

Univariate analysis showed that different ages, education levels, monthly family incomes, marital relationships, gender expectations, gestational weeks, and whether the pregnancy was planned or unplanned, were factors affecting the sleep quality of women pregnant with a second child (*P* < 0.05), see Table 3.

**Table 3:** Univariate analysis of factors affecting the sleep quality of second-born pregnant women [*n* (%)]

Factors	Number of cases	Sleep disorder [*n*, (%)]	Normal sleep [*n*, (%)]	*X*^2^ value	*P* value
Age				4.117	0.042
< 30 years	66	31 (46.97)	35 (53.03)		
≥ 30 years	96	30 (46.97)	66 (46.97)		
Occupation				0.607	0.738
Civil service and institutions	72	25 (34.72)	47 (65.28)		
Enterprises and workers	58	24 (41.38)	34 (58.62)		
Self-employment	32	12 (37.50)	20 (62.50)		
Education level				9.500	0.023
Junior high school and below	5	3 (60.00)	2 (40.00)		
High school or technical secondary school	39	22 (64.10)	17 (35.90)		
College or undergraduate	104	32 (30.77)	72 (69.23)		
Postgraduate and above	14	4 (28.57)	10 (71.43)		
Family monthly income				10.304	0.016
< 3000 yuan	6	4 (66.67)	2 (33.33)		
3000–5000 yuan	25	14 (56.00)	11 (44.00)		
5000–10,000 yuan	55	23 (41.18)	32 (58.82)		
> 10,000 yuan	76	20 (26.31)	56 (73.69)		
Medical payment mode				0.491	0.782
Rural Cooperative Medical System	44	18 (40.91)	36 (59.09)		
Provincial medical insurance or municipal medical insurance	78	27 (34.62)	51 (65.38)		
At one's own expense	40	16 (40.00)	24 (60.00)		
Husband and wife relationship				5.964	0.015
Satisfied	143	49 (34.27)	94 (65.73)		
Just so so	19	12 (63.16)	7 (36.84)		
Family support (parents)				0.390	0.532
Very supportive	134	49 (36.57)	85 (63.43)		
Just so so	28	12 (42.86)	16 (57.14)		
Fetal gender expectation				7.379	0.007
Yes	84	40 (47.62)	44 (52.38)		
No	78	21 (26.92)	57 (73.08)		
Planned pregnancy or not				5.338	0.021
Out of plan	82	38 (46.34)	44 (56.66)		
Within the plan	80	23 (28.75)	57 (71.25)		
Number of pregnancies				0.203	0.904
2 times	92	36 (39.13)	56 (60.87)		
3 times	34	12 (35.29)	22 (64.71)		
> 3 times	36	13 (36.11)	23 (63.89)		
Pregnancy complications				1.153	0.283
Yes	16	8 (50.00)	8 (50.00)		
No	146	53 (36.30)	93 (63.70)		
History of miscarriage				0.276	0.599
Yes	86	34 (39.53)	52 (60.47)		
No	76	27 (35.53)	49 (64.47)		
Gestational week				6.331	0.042
Early pregnancy (≤ 12 weeks)	11	3 (27.27)	8 (72.73)		
Second trimester (13–27 weeks)	46	14 (30.43)	31 (69.57)		
Third trimester (≥ 28 weeks)	105	44 (41.90)	61 (58.10)		

### Multivariate logistic regression analysis of factors affecting the sleep quality of women pregnant with a second child

Multivariate logistic regression analysis showed that low education levels, low family monthly income, gender expectations, and poor marital relationships are the main factors affecting pregnant women’s poor sleep quality (see Table 4).

**Table 4:** Logistics regression analysis of related factors affecting the sleep quality of second-born pregnant women (*n* = 162)

Entering variable	*b*	*S*_b_	Waldχ^2^	*P*	OR	95%CI
Husband and wife relationship	1.296	0.374	12.064	0.001	0.275	0.132–0.568
Education level	1.862	0.718	6.682	0.011	0.224	0.052–0.964
Family monthly income	1.112	0.465	5.987	0.014	3.035	1.251–7.398
Fetal gender expectation	2.374	1.345	4.132	0.038	0.065	0.005–0.908

## Discussion

The results showed that, among the 162 women pregnant with a second child, 61 had sleep quality problems (PSQI total score > 7 points), accounting for 37.65% (61/162). This study showed that the sleep quality of women pregnant with a second child is poor, and the percentage is higher than that of normal female [16]. Nguyen et al. [17] found that the sleep quality of multipara was worse than that of nullipara. The study of Christian et al. [18] showed that compared with nullipara, the multipara experienced worse sleep qulity in the early stage of pregnancy, which was consistent with the results of this study. Adequate sleep can increase the secretion of lymphocytes, IL-6, and other substances in pregnant women, thereby improving their immune function [19]. Insufficient sleep or poor quality sleep can lead to various physical problems [20–22], such as: (1) endocrine dysfunction, (2) decreased pituitary growth hormone secretion, and (3) disordered secretion of serotonin 1A receptor and dopamine D2 receptor in different regions of the brain. The improvement of immune function is particularly important for the healthy of pregnant women and the healthy growth and intellectual development of fetus. In 2015, the Standing Committee of the Twelfth National People’s Congress passed the amendment to the Population and Family Planning Law, advocating that a couple can have two children. With the full liberalization of China’s two-child policy, the number of pregnant women with two children will increase significantly [9]. Therefore, paying attention to the sleep quality of women pregnant with a second child is a vital part of health care.

The results of this study also showed that low education levels, low family monthly incomes, gender expectations, and poor marital relationships are the main factors for the sleep quality of women pregnant with a second child. Zhang’s study [23] showed that monthly income (*β* = 1.469, 95%CI 1.135, 3.172), unplanned pregnancy (*β* = 1.786, 95%CI 1.002, 2.732) and the relationship between mother-in-law and daughter-in-law (*β* = 1.398, 95%CI 1.046, 2.132) were risk factors affecting sleep quality. Zheng Xiali [24] showed that education level (OR = 0.425, 95%OR: 0.253, 0.705), monthly income (OR = 1.842, 95%OR: 1.063, 3.167), fetal gender expectation (OR = 2.361, 95%OR: 1.294, 4.216) and conjugal relation (OR = 1.176, 95%OR: 1.018, 1.365) were the main factors affecting the sleep quality of pregnant women. These were similar to the results of this paper. Women pregnant with a second child with low education levels and low family incomes have relatively little objective support, and the financial burden during pregnancy is heavier. They are worried about the cost of raising their children after delivery, leading to sleepless nights. However, most women with a second pregnancy have substantial work themselves. Besides being busy with jobs, they also have to take on more housework in the family. There is nowhere to vent their life and physical troubles, and they cannot get enough rest. Their satisfaction and happiness are greatly reduced, affecting the quality of sleep. The women pregnant with a second child with a higher education level will seek more health care knowledge and have more resources to understand the relevant protection measures for the safety of the fetus and themselves. Gender expectation is also one of the influencing factors for the poor sleep quality of women pregnant with a second child. Due to the influence of traditional culture on pregnant women and their families, some will want to have boys or expect to have girls. This expectation and the increase in the cost of raising a child in the future will bring tremendous pressure to pregnant women and may cause them to be stressed for a long time. In a restless mood, it is difficult to fall asleep. At the same time, if the relationship between husband and wife is not good, there may be less support from the husband, which easily leads to poor sleep quality for pregnant women. The possible reason is that the concept of family for the majority of Chinese people is a small family consisting of a monogamous marriage in a narrow sense, and siblings who have become independent after marriage are classified as relatives outside the family. For pregnant women, the husband is the primary source of material and spiritual support necessary for the family, and support from relatives outside the family is limited. If there is less support from the husband and the family atmosphere is not harmonious or unstable, pregnant women will not be able to get enough sense of security and are more likely to stimulate serotonin receptor regulation disorders in the brain, which affects the quality of sleep [25].

This study has some limitations: (1) the sample size was small; (2) the study was only conducted in a single region, which may have regional limitations. Large sample multicenter studies can be conducted later; and (3) the study was influenced by domestic fertility policy, so it may have cultural limitations.

## Conclusions

In summary, low education level, low monthly family income, gender expectation, and poor marital relationship are the dominant factors of insufficient sleep quality in pregnant women with second birth. Special attention should be paid to the sleep quality and health status of women with the above risk factors in the second pregnancy, and targeted intervention measures should be taken to carry out psychological guidance and sleep health education during pregnancy to improve the health care level of pregnant women.

## Data Availability

All data generated or analyzed during this study are included in this published article.
